# Flavonoids and Other Phenolic Compounds for Physiological Roles, Plant Species Delimitation, and Medical Benefits: A Promising View

**DOI:** 10.3390/molecules29225351

**Published:** 2024-11-14

**Authors:** Sompop Kuljarusnont, Satoshi Iwakami, Tsukasa Iwashina, Duangjai Tungmunnithum

**Affiliations:** 1 Department of Obstetrics and Gynecology, Faculty of Medicine Siriraj Hospital, Mahidol University, Bangkok 10700, Thailand; sompop.kul@mahidol.edu; 2 Graduate School of Agriculture, Tokyo University of Agriculture and Technology, Fuchu 183-8509, Tokyo, Japan; iwakamis@go.tuat.ac.jp; 3 Department of Botany, National Museum of Nature and Science (TNS), Tsukuba 305-0005, Ibaraki, Japan; iwashina@kahaku.go.jp; 4 Department of Pharmaceutical Botany, Faculty of Pharmacy, Mahidol University, Bangkok 10400, Thailand; 5 Le Studium Institute for Advanced Studies, 45000 Orléans, France

**Keywords:** flavonoids, phenolics, plant secondary metabolites, physiological roles, plant species delimitation, medical application

## Abstract

Flavonoids and other phenolic constituents are a large group of plant metabolites that have long attracted interest from researchers worldwide due to their functions in plant physiology, as well as their huge number of benefits for human health and well-being. This review attempts to reveal a promising view of the major physiological roles of flavonoids and other phenolic phytochemical molecules, e.g., protection agents against UV damage, pathogen defense agents, detoxifying agents, and agents promoting pollen fertility and successful pollination. Besides, the value of both flavonoids and other phenolic phytochemicals for plant species delimitation was also emphasized for the first time with the determination of their major physiological roles. Furthermore, their medical benefits for mankind were also highlighted in this current work.

## 1. Introduction

Plant secondary metabolites, or so-called phytochemical compounds, are molecules produced by plants which are not directly crucial for basic functions such as photosynthesis or respiratory metabolism, but are required for the better survival of plants in various environmental conditions [1,2,3,4]. Among them, plant phenolic substances, including flavonoids, are one of the largest groups of phytochemical compounds and have been widely employed in a huge number of research works, with an increasing trend in interest from researchers in order to understand both the functions and potential applications of these phytochemical compounds in various fields [3,5,6,7,8,9,10,11,12,13,14]. In addition, these secondary metabolites are produced in almost every part of the living organisms in the Plantae kingdom [15,16,17,18,19,20], so the great benefits of these substances cannot be denied.

In addition, these flavonoids and other phenolic compounds are also useful for plant species delimitation to distinguish different species from other closely related ones. Normally, plant taxonomic works begin with using morphological characteristics to delimit plant species, as this is simple and saves analysis costs [21,22,23,24,25]. However, some closely related species of plants consist of many similar morphologies or overlapping morphological characteristics that cannot be used for identification at the species level using morphological evidence, such as the five sister species of Peruvian chili peppers from the *Capsicum* genus [26]. In addition, some groups of plants have very many morphological variations, which provides such a great variation in the morphological characteristics of sister species or complex species, e.g., the three *Phyteuma* species members from the Campanulaceae family, that they cannot be classified and identified by morphological data [27]. Accordingly, the phytochemical profiles of these plants, especially their flavonoids and other phenolic constituents, are also helpful for delimiting these species [26,27].

The aim of this review is to illustrate the importance of these phytochemical compounds regarding their physiological roles, such as protective agents against UV damage, pathogen defense agents, detoxifying agents, and agents promoting pollen fertility, successful pollination, and plant species delimitation, as well as medical benefits for mankind. So, key words were searched in Google scholar, Scopus, and PubMed in order to obtain publications on the targeted topic, and 785 publications related to the key words were found. Then, these publications were carefully read so as to find recent and non-redundant research studies that met the aim of this review. Some older publications to highlight some essential points were also included in this work.

## 2. Biosynthesis of Flavonoids and Other Phenolic Compounds (Figure 1)

Phenolic compounds, comprising several types of flavonoids, are commonly known as a major group of natural secondary metabolites synthesized by plants themselves. These plant phenolic components are synthesized from phosphoenolpyruvate erythrose-4-phosphate using the shikimate and phenylpropanoid pathways [15,28,29] to deliver phenylpropanoids directly, and they are also produced via acetyl-Co A using the acetate–malonate pathway [28,29] to provide simple phenols. Hydroquinone-*O*-beta-D-glucopyranoside, or arbutin, is a concrete example of a simple phenol compound, which can be found in the leaves of many species of both berry and pear [7]. In general, the metabolism of phenylpropanoids delivers a series of hydroxycinnamic acids, e.g., 5-*O*-caffeoylquinic acid and chlorogenic acid. These phenolics are found not only free-form, but also combined with sugars as glycosides in various families of eudicot, such as Rosaceae, Rubiaceae, Apiaceae, Asteraceae, Fabaceae, Moraceae, and Solanaceae [29,30,31,32].

**Figure 1:**
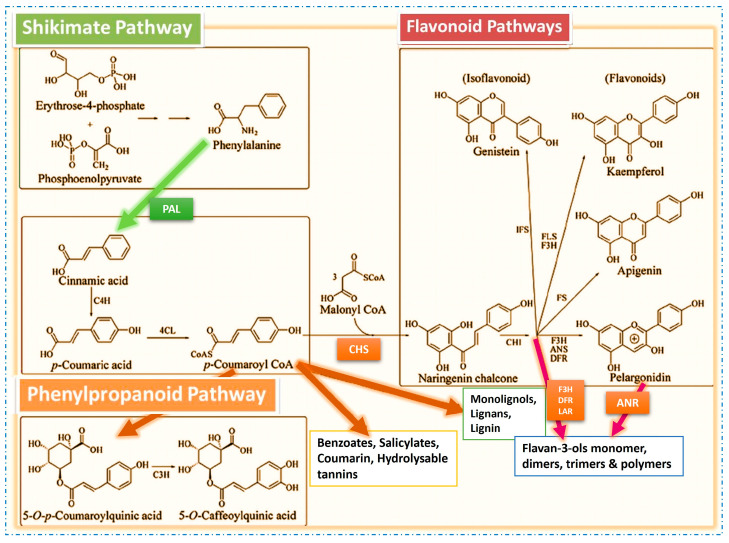
Major biosynthesis pathways of flavonoids and other phenolic compounds. PAL = phenylalanine ammonia-lyase; C4H = cinnamate-4-hydroxylase; 4CL = 4-coumaroyl: CoA-ligase; HCT = hydroxycinnamoyl transferase; C3H = p-coumarate-3-hydroxylase; CHS = chalcone synthase; CHI = chalcone isomerase; ANS = anthocyanidin synthase; DFR = dihydroflavonol reductase; FS = flavone synthase; FLS = flavonol synthase; F3H = flavanone 3-hydroxylase; IFS = isoflavone synthase; ANR = anthocyanidin reductase; LAR = leucoanthocyanidin reductase.

The carbon taken in from carbon dioxide to produce malonate is similar to the carbon released in the condensation/decarboxylation reaction catalyzed by CHS enzymes (chalcone synthase or naringenin-chalcone synthase). The other selective enzymess that also work in this process include chalcone isomerase, anthocyanidin synthase, dihydroflavonol reductase, flavone synthase, flavonol synthase, flavanone 3-hydroxylase, isoflavone synthase, and so forth. These complicated pathways produce monomeric and polymeric compounds which accomplish various tasks in plant physiology [4,15,16,26,27,28,33,34,35,36,37,38,39,40,41,42,43,44,45,46,47,48,49,50,51]. Plant phenolic substances exhibit extremely diverse groups with a particularly large structural diversity [1].

**Figure 2:**
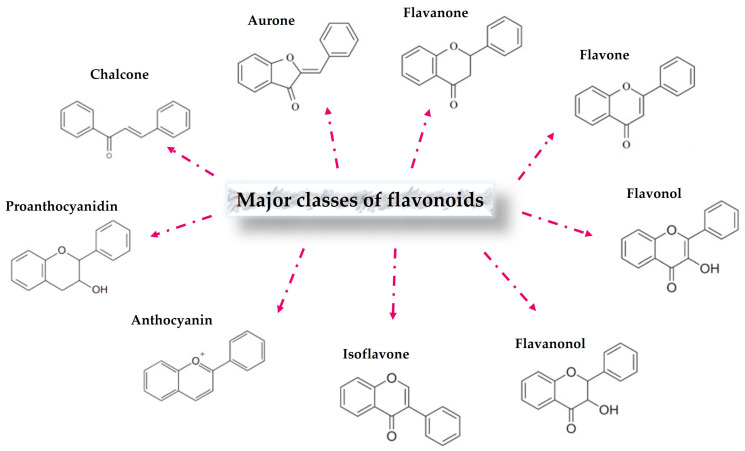
Chemical structures of the major classes of flavonoids.

Many reports have indicated that flavonoids are one of the biggest groups of phytochemical products, and over 10,000 structures of flavonoids have been identified [52,53,54]. Flavonoids can be found in various plant tissues, especially photosynthetic tissue such as mesophyll layers [3]. They are distributed not only inside the cell, but also throughout the surfaces of many organs or tissues [1,3]. The flavonoid chemical structure is mainly based on a C6-C3-C6 skeleton, depending on the position of the linkage between the aromatic ring and benzopyrano moiety. They naturally occur in both forms of aglycones and glycosides, depending on the plant species and environmental factors, and involve various classes [53], for example, flavones, isoflavones, flavanones, flavonols, flavanonol, aurones, chalcones, and anthocyanin (Figure 2).

In *Arabidopsis thaliana*, a plant model, the regulation of the flavonoid biosynthetic pathway has been determined. The flavonoid biosynthesis of *Arabidopsis* is regulated by single copy genes which encode all enzymes of the central flavonoid metabolism, excluding flavonol synthase (FLS). This enzyme is encoded by six genes, but only the activities of the FLS1 and FLS3 genes have been proven [55,56]. According to the report of Cheynier and her research team, they found that flavonoid production is affected by the transcription rate of the flavonoid biosynthetic genes [1]. Many kinds of transcription factors (TFs) play necessary roles in flavonoid biosynthesis, and some of them are conserved among monocotyledon and dicotyledon [51,57,58,59]. A concrete example is anthocyanin and proanthocyanidin biosynthetic genes, which consist of specific members of the R2R3-MYB and basic helix–loop–helix transcription factor families combined with a WD-repeat protein [60]. Likewise, 3-deoxyflavonoids and flavonol biosynthesis in several grass species are controlled by transcription factor family genes, *R2R3-MYBs* [50].

## 3. Major Physiological Functions of Flavonoids and Other Phenolic Compounds

### 3.1. Protective Agent Against UV Damage

From the point of view of protection against ultraviolet (UV) radiation damage, flavonoids and other phenolic compounds played a key role in assisting embryophytes and land plants to adapt and evolve themselves from an aquatic to a terrestrial environment in the middle of the Palaeozoic era between 480 and 360 million years ago. At that time, the original terrestrial plants such as mosses, liverworts, hornworts, and some tracheophytes were able to synthesize various phenolic substances so as to address specific requirements, and in particular, these substances acted as UV light screens to help land plants to survive in such an extremely changed environment [5,6,8,34,61].

The spectra of UV radiation can be divided into lower energy UV-A (320–400 nm), higher energy UV-B (280–320 nm), and UV-C (254–280 nm). The most severe damage is caused by the UV-B band, which may affect the chlorophyll pigments in chloroplasts, proteins, the phospholipid bilayer of cell membranes, photosynthesis, transpiration, and pollination [50,62,63,64,65]. The higher the altitude, the higher the intensity of UV radiation. According to this, terrestrial plants, especially alpine species, protect themselves by using flavonoids and other phenolic compounds [62]. Flavonoids have the helpful abilities of absorbing harmful UV light and scavenging various types of free radicals in plant cells and tissues [66].

Murai and his research team surveyed both quantitative and qualitative variations of UV-absorbing flavonoids in the leaves of *Fallopia japonica* [currently, this species is synonymous with *Reynoutria japonica* Houtt., https://www.worldfloraonline.org/taxon/wfo-0000406106#distributionMap, searched on 1 November 2024] and the needles of *Larix kaempferi* (Lamb.) Carrière, which grew at various altitudes on Mt. Fuji in Japan. They found an increase in flavonoids content in the leaves of *F. japonica* and the needles of *L. kaempferi* when the altitudes of their habitats were increased [66]. Likewise, a relationship between altitudinal variation and UV-absorbing flavonoids has also been reported in *Plantago asiatica* [67] and Himalayan *Rheum nobile* Hook. f. & Thomson [68]. Phytochemical studies have reported that B-Ring ortho-dihydroxylated flavonoids are also synthesized by highland plants that live at high altitudes, e.g., *Plantago asiatica*, *Geum calthifolium* var. *nipponicum*, and *Sieversia pentapetala*. Seashore *Calystegia soldanella* (L.) R. Br. populations exhibited chemical adaption trends to survive the environmental stress posed by UV irradiation by accumulating quercetin glycosides [67] in order to protect themselves from intense UV-B conditions.

### 3.2. Pathogen Defense Agent

Phenolic compounds play an essential role in plant defense and repair mechanisms, including pathogen defense. Plants cells and tissues accumulate phenolic compounds by increasing the activities of enzymes such as phenylalanine ammonialyase, chalconesynthase, and phosphoenolpyruvate-carboxylase when they are infected by pathogens [49,69,70]. Phytoalexins are one of the major groups of natural phytochemicals that interact with the pathogen defense processes, which are low-molecular-weight antimicrobial components that are synthesized and accumulated in plant cells and tissues after infection by bacteria, viruses, or other pathogens [1]. Several phenolics, including flavonoids such as isoflavonoid glycosides, have been classified as important precursors for phytoalexin production during infection [28].

Moreover, the antipathogenic properties of several flavonoids can be non-specific, resulting from their antioxidative properties [48,71,72]. Flavonoid compounds help to reduce reactive oxygen species that are generated by plants and pathogens through infection [73,74]. Flavonoids are transported to the infection sites of individual plants and induce the hypersensitivity reaction, then cause programmed cell death and also combine with the cell walls of necrotic and adjacent cells [47,73,74]. Additionally, these substances can prevent pathogen infection by modulating auxin (IAA) activity, resulting in tissue differentiation, calluses, and tylose formation [47]. According to the study of Treutter in 2005 on the flavonoids involved in plant resistance, he found that flavonoids may directly cause pathogen enzyme inhibition by chelating metals that are required for the pathogen’s activity [46,75,76].

### 3.3. Detoxifying Agent

A major source of active oxygen species is the photosynthetic electron transport chain, so chloroplasts have evolved a detoxification system to avoid oxygen-mediated toxicity [4,45,77,78,79]. Flavonoids play a significant role as detoxification agents in these processes. Yamasaki and his research team demonstrated that, if flavonoids are localized, super oxide anion radicals cannot freely diffuse into the vacuoles of plant cells. They reported that the flavonoid–peroxidase reaction acted as a detoxification factor against H_2_O_2_ in plant cells such as *Schefflera arboricola* Hayata [currently, this species is synonymous with *Heptapleurum arboricola* Hayata, https://www.worldfloraonline.org/taxon/wfo-0000981504, searched on 1 November 2024] [44]. Moreover, Jansen and colleagues conducted research on plant stress from ultraviolet radiation and pointed out that phenolic compounds were efficient scavenging agents for reactive oxygen species [43]. Phenolics may also help to inactivate iron ions by chelation and additional suppression via the super oxide-driven Fenton reaction.

Furthermore, flavonoids and phenolic compounds are also necessary for antioxidant activity in the electron transport pathways inside plant mitochondria [77]. There are two electron transport pathways in mitochondria, including the cytochrome and alternative pathways. Results from a study by Shimoji and Yamasaki showed that the flavonoids myricetin, quercetin, and kaempferol displayed an antioxidant property that could inhibit alternative oxidase activity [42]. Additionally, ortho-dihydroxy B-ring-substituted quercetin and luteolin were accumulated in the leaves of *Ligustrum vulgare* L. in mesophyll and epidermal tissues, and it was found that these ortho-dihydroxy B-ring-substituted flavonoids protected plant tissues from oxidative damage from sunlight [41]. In grapevine leaves (*Vitis vinifera* L.), the flavonoid redox cycle was investigated as an alternative system for detoxification against H_2_O_2_ [80].

### 3.4. Pollen Fertility and Successful Pollination

Flavonoids and other phenolics are synthesized in almost every part of a plant, and these phytochemical compounds play necessary roles in providing color, fragrance, and taste to flowers, fruits, and seeds, which are important factors for attracting pollinators and seed dispersers [37,81,82,83]. Yellow pollen grains with various ranges of visible and ultraviolet reflection spectra, which can be detected by the targeted animal pollinators, are produced from the unique microspore mother cells and combinations of different flavonoids in each plant species, and this leads to successful pollination [37,84]. Many studies have reported that flavonoids are used by plants to produce the distinct yellow color of pollen grains, but white-colored pollen has also been described in maize, morning glory, and petunia [40,83,85,86].

In 1992, Mo and colleagues conducted research on the white pollen grains of maize and petunia to examine the role of flavonols in functional pollen via chalcone synthase mutants. This was the first report on the correlation between flavonoids and the fertility of pollen grains. This research team discovered that the mutant groups exhibited deficiencies in flavonoids and sterile pollen because these pollen grains could not produce functional pollen tubes. Interestingly, after adding kaempferol (the flavonol class of flavonoids) during pollination, the pollens of these mutant plants could grow functional pollen tubes similar to those of wild types [85]. Moreover, they found that flavonoid-deficient pollen cannot self-cross via its own stigma, but can function partially on the stigma of wild-type plants which contain flavonol substances [85]. Pollak and his team worked on *Petunia hybrida* E.Vilm. and investigated the effects of chalcone synthase and flavonol accumulating on the stigmas and anthers of this species. They generated flavonoid-deficient mutants that lacked chalcone synthase to determine the important roles of flavonoids in pollen fertility [87].

In tomato (*Solanum lycopersicum* L.), the RNA interference silencing of the chalcone synthase gene, which participates in the first step of flavonoid biosynthesis, led to parthenocarpic tomato fruits [38]. The post-transcriptional silencing of the FLS gene of *Nicotiana tabacum* L. also resulted in a decrease in seed production [39]. In addition, the silenced mutant lines exhibited lower levels of flavonol and anthocyanidins, whereas the flavan-3-ol level was increased. Flavonols, particularly quercetin, demonstrated their essential roles in pollen tube germination and successful plant pollination, not only in in vitro, but also in in vivo experiments [39].

Besides, flavonoids and other phenolic compounds in plant physiological functions from various plant species were provided to give examples on this perspective (Table 1).

## 4. Flavonoids and Other Phenolic Compounds vs. Plant Species Delimitation

During its evolution, the extreme biodiversity of the plant kingdom has generated not only species diversity, but also a variety of flavonoids and other phenolic components. Additional analyses of different plant species will lead to the discovery of novel structures and possibly new metabolic pathways that provide new plant systematic data to determine plant taxonomic statuses and species boundaries [25,27,36,84,88,89,90]. In plant biosystematics, many types of information, such as classical morphological, phenetics (morphometric or numerical taxonomy), anatomical, palynological, chromosome, DNA sequencing, and phylogenetic analysis data, have been combined and analyzed together, so as to provide a concrete conclusion on the species delimitation of several plants. However, large numbers of plant taxa are still waiting for further information to clearly delimit their species boundaries. Likewise, a research team from Peru and France studied Peruvian chili peppers from the *Capsicum* genus belonging to the Solanaceae family, which display overlapping morphological characteristics, so these *Capsicum* species, particularly *Capsicum baccatum*, *Capsicum chinense*, and *Capsicum frutescens*, fail to use classical taxonomy for their species delimitation [26]. The results from this research team pointed out that their flavonoids and other phenolic compounds were the main biomarkers that were helpful to delimit these *Capsicum* species members at the species level [26]. Additionally, a European research team from Slovenia conducted research on the species members of the *Phyteuma* genus belonging to the Campanulaceae family, which have many variations in morphological traits [27]. This great variation has led to confusion and misinterpretation in using taxonomic keys that are constructed by using morphological characteristics. Their results proved that phenolic compounds are useful biomarkers for the species delimitation of taxonomic complexes in the *Phyteuma* genus at both specific and intraspecific levels [27].

Some plant taxa synthesize specific flavonoids and/or phenolics, e.g., legume families produce isoflavonoid substances. Moreover, *Sorghum bicolor*, *Sinningia cardinalis*, and *Zea mays* [36] are a few species that commonly synthesize 3-deoxyanthocyanins [36]. An obvious example is the species members in *Viteria*, a species-rich genus (Dipterocarpaceae family). Using flavonoid analysis, the *Vatica* genus was reported to have 65 species distributed throughout Asian countries [91]. Joshi conducted a chemotaxonomic study on three species members that still have species delimitation conflicts, and her results pointed out that quercetin 3-glucoside, apigenin 5-glucoside, kaempferol 3,5-glucoside, and quercetin 3-rutinoside can be used as chemotaxonomic markers to delimit these studied species [92]. Furthermore, the patterns of flavonoid aglycones and glycosides have also been reported as useful tools for the species delimitation of species members in the Shorea Roxb. ex Gaertn genus [35,93].

Similarly, Clark and Mabry studied Hazardia species and demonstrated that the glycosides of quercetin, kaempferol, isorhamnetin, luteolin, and apigenin, the glycoflavone of apigenin, and methoxylated flavonol aglycones were helpful for species delimitations of *Hazardia* species, together with additional information on morphology and geography [94]. The taxonomic problem in the species delimitation of the Agave (Agavaceae) genus was also solved by chemotaxonomic data on phenolics [95]. In addition, Chin-Sung and his Korean research team focused their study on the *Adonis amurensis* complex distributed in eastern Asia. Excessive morphological variations in the flower led to taxonomic problems with species boundaries and confusion about species definition and recognition for further economic applications [96]. This team isolated and characterized 19 flavonoids and phytochemical compounds from the leaves and flowers of the *A. amurensis* complex; they proposed that it was probably an evolutionary advancement that affected the loss of some C-glycosylflavones and O-glycosylflavone synthesis in this complex species [96]. These studies also suggest that flavonoids and phytochemicals can be as variable in plant species delimitation as morphological characteristics [94,95,96]. Additionally, phenolic compounds were also reported as useful evidence for species delimitation in Tamaricaceae species members by a Russian research team [97]. This team determined the phenolic phytochemical profiles of poorly studied Siberian species belonging to the *Myricaria* Desv. genus, such as *Myricaria longifolia* (Willd.) Ehrenb. and *M. bracteata* Royle [97].

**Figure 3:**
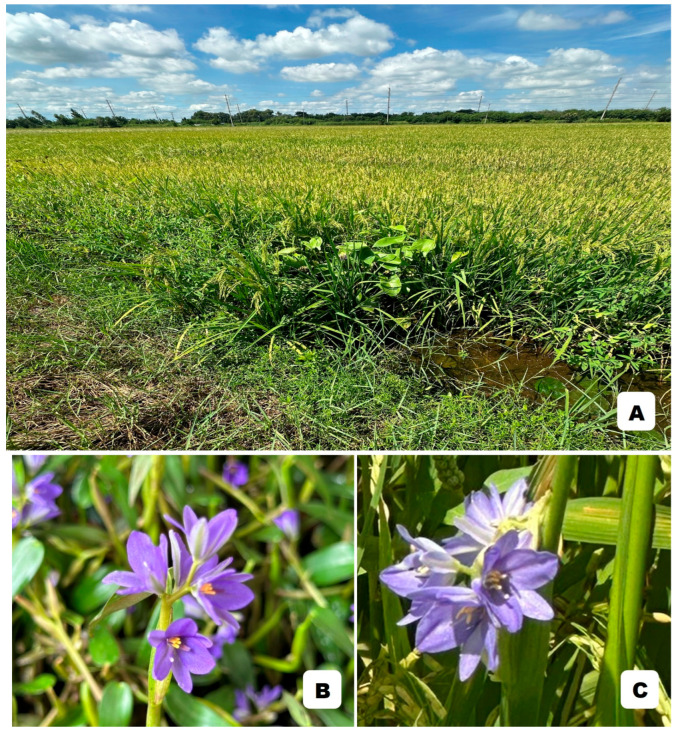
*Monochoria hastata* (L.) Solms and *Monochoria angustifolia* (G. X. Wang) Boonkerd & Tungmunnithum grow in the same natural habitat such as rice field. (**A**) Natural habitat: rice field in Thailand (**B**) inflorescence of *M. angustifolia*, and (**C**) inflorescence of *M. hastata*. All photos were taken in Thailand in August 2024 by Assoc. Prof. Dr. Duangjai Tungmunnithum.

Besides terrestrial plants, the usefulness of flavonoids and other phenolic compounds for plant species delimitation has also been reported for aquatic plant species [14,88,98,99]. *Monochoria hastata* (L.) Solms and *M. angustifolia* (G. X. Wang) Boonkerd & Tungmunnithum, aquatic flowering plants that are species members of the *Monochoria* C. Presl genus (Pontederiaceae family), have been used not only as local vegetables, but also as ingredients in traditional medicine recipes [88,98,99]. *M. hastata* and *M. angustifolia* are distributed in Thailand and some countries in Asia, and they mostly grow in the same natural aquatic habitats (Figure 3), such as rice fields and other water bodies [88]. The high variation in the morphological characteristics of both their vegetative and reproductive parts has caused confusion in their taxonomic status, as well as their species boundaries. Research studies were conducted on almost 500 plant samples from these *Monochoria* species, covering populations distributed throughout all the floristic regions of Thailand. The results revealed that the flavonoids’ phytochemical profile was useful for investigating the evolutionary connections of these *Monochoria* species members, as well as for the botanical authentication of these two medicinal plant species [88].

Another example was investigated in *Nelumbo nucifera* Gaertn. and *Nymphaea lotus* L., which are aquatic medicinal plants that can be found in the same aquatic habitats [14,100]. Dried and/or powdered stamens of *N. nucifera* are widely used as a valuable raw plant material for various kinds of traditional medicines [14,100]. However, dried and/or powdered stamens of *N. nucifera* are frequently confused with those of *N. lotus*, and this adulteration leads to issues with the costs of raw material and the efficacy of phytopharmaceutical products for industrial sectors [14]. The results from this research work illustrated that flavonoid phytochemical compounds provide potential evidence for solving authentication problems and identifying *N. nucifera* and *N. lotus* from their dried stamen materials [14].

## 5. Medical Benefits of Flavonoids and Other Phenolic Compounds

Besides their physiological functions in plants as valuable secondary metabolites, flavonoids and other phenolic phytochemical compounds also play key roles in offering various medical benefits for mankind to promote human health and well-being [11,12,13,30,31,100,101,102,103,104,105,106,107,108,109,110,111]. In this current decade, a huge number of research teams worldwide are conducting research on the potential of these flavonoids and other phenolic bioactive molecules for both the treatment and prevention of several diseases [12,32,88,103,105,112,113,114,115,116,117,118,119,120,121,122,123,124,125]. In addition, in commercial sectors such as phytopharmaceuticals, cosmetics, and cosmeceuticals, companies are also conducting research on these phytochemical compounds to seek information on their potential bioactive molecules as major ingredients for product development [30,101,109,110,126,127].

The potential of dihydromyricetin, a major flavonoid phytochemical compound from *Ampelopsis grossedentata*, for diabetic cardiomyopathy was evaluated by Chen and his research team from China [102]. This team worked on a diabetic mice model and used a treatment group administered with dihydromyricetin 250 mg/kg/day for 12 weeks [102]. The results showed that this flavonoid helped to improve cardiac dysfunction, fibrosis, and injury; this bioactive molecule also reduced inflammation, oxidative stress, and necroptosis via sirtuin 3 (SIRT3) activation in a streptozotocin-induced diabetic mice model [102]. Vázquez-Ruiz and a team from Spain researched the prevention of cardiovascular problems via the rich intake of phenolics from red wine, olives, and olive oil in the Mediterranean diet [103]. This research team followed 16,147 Spanish participants, who were the young Mediterranean cohort for this project, for more than 12 years using a 136-item questionnaire to validate food consumption frequency, and their polyphenol intake was obtained by using the Phenol-Explorer database. This study pointed out that a moderate-to-high dietary consumption of phenolic phytochemical compounds, especially flavonoids, may possibly decrease the incidence of cardiovascular disease by the means of the Mediterranean dietary pattern [103]. Phan and his group determined the medical potential of a flavonoid-rich extract from *Eclipta prostrata* L., which is traditionally used for enhancing memory and cognitive functions and diabetes management [104]. This study illustrated that the flavonoid-rich extract from this plant moderately inhibited α-glucosidase and α-amylase and provided high antioxidant and anti-acetylcholinesterase effects; the molecular docking results also showed that most of the identified flavonoids and other phenolic major compounds exhibited good ADMET properties [104].

The antibacterial and antifungal effects of *Phoenix dactylifera* L. from the Arabian desert with a leaves extract rich in flavonoids and phenolic acids were tested [11]. Silver nanoparticles were green-synthesized by ethanolic and water extracts from *P. dactylifera* leaves; the antifungal activity was tested in different species of *Candida*, and the antibacterial activity was assessed in both two Gram-positive and two Gram-negative strains [11]. This team found that silver nanoparticles had noteworthy antimicrobial effects, whereas the water extract provided antimicrobial activity a little higher than that of the ethanolic extract. So, the researcher suggested that palm leaf extracts fight against pathogenic bacteria and also fungi, instead of using chemical substances [11]. Another study focusing on the antibacterial, antifungal, insecticidal, and phytotoxic potential of a flavonoid-rich extract was conducted by a team from Pakistan and Saudi Arabia, and they studied silver nanoparticles from *Amaryllis vittata* (L.) Herit leaf and bulb extracts [30], as well as French pine bark extract chewing gum [101]. The results from these findings demonstrated the potential for developing new interesting agricultural applications and pharmacological/medical products [30,101].

The healthy benefits of flavanol-rich cocoa have also been evaluated by various research teams [109,110]. Davinelli and his team from Italy and the USA conducted a randomized controlled trial to evaluate the potential of flavanol-rich cocoa in 48 healthy human subjects for 4 weeks of flavanol-rich cocoa supplementation, comparing them with the baseline. The results supported the health benefits of flavanols from cocoa [109]. Another randomized clinical trial involving 22 healthy participants was conducted on the flavanols from dark chocolate by a research team from Germany [110], who used a double-blind crossover clinical trial analysis with participants consuming 20 g of dark chocolate (comprising 400 mg of flavanols) or 7.5 g of milk chocolate. This research proposed the medical benefits of vasodilating flavanols from dark chocolate on visual function [110]. Another study by Cádiz-Gurrea and his research group from Peru and Spain focused on qualitatively determining the flavonoids and other phenolic phytochemical compounds in husk and bean (cocoa by-products) extracts from different cocoa-growing areas in Peru using high-performance liquid chromatography (HPLC) coupled with mass spectrometry, also emphasizing the potential of cocoa by-products as high added-value products for medical, nutraceutical, and pharmaceutical manufacturing [111].

## 6. Conclusions

It can be clearly seen that flavonoids and other phenolic components play significant roles in various physiological activities, such as protective agents to fight against damage from ultraviolet (UV) radiation, pathogen defense agents, and detoxifying agents in order to help several plants to survive in challenging environments with various biotic and abiotic factors. In addition, these phytochemicals also help to promote pollen fertility and successful pollination, which are the most important processes for conserving their species. Nonetheless, these secondary metabolites are also effective tools for plant species delimitation and classification depending on the group of plants. Therefore, additional studies and surveys of flavonoids and other phenolics from new/undiscovered plant species need to intensively investigate in order to make progression in these research areas and achieve a better understanding of plant physiology and evolution. Additionally, these phytochemical compounds also offer an immense benefit to human beings, following a huge number of research studies performed to determine their medical advantages in in silico, in vitro, and in vivo animal models, as well as many clinical trials. Nevertheless, long-term studies, toxicity tests, and greater numbers of participants (subject size) for many clinical trials should be implemented to ensure the safety and efficacy of these flavonoids/phenolic-rich supplements, phytopharmaceutical products, and medicines.

## Figures and Tables

**Table 1:** Flavonoids and other phenolic compounds in plant physiological functions.

Plants	Native or Cultivated Regions	Major Flavonoids/Other Phenolic Compounds	Physiological Functions	Reference
*Reynoutria japonica* Houtt.	Japan	Flavonol 3-*O*-glycosides	UV-absorbing agent	[66]
*Larix kaempferi* (Lamb.) Carrière	Japan	Flavonol 3-*O*-glycosides	UV-absorbing agent	[66]
*Calystegia soldanella* (L.) R. Br.	Japan	Kaempferol 3-*O*-rutinoside, 3-*O*-glucoside and 3-*O*-rhamnoside, quercetin 3-*O*-rutinoside, 3-*O*-glucoside, 3-*O*-rhamnoside and 3-*O*-apiosyl-(1 → 2)-[rhamnosyl-(1 → 6)-glucoside]	UV-absorbing agent	[67]
*Rheum nobile* Hook. f. & Thomson	Japan	Quercetin 3-*O*-glucoside, quercetin 3-O-rutinoside, quercetin 3-*O*-galactoside, quercetin 3-*O*-arabinoside and quercetin 3-*O*-[6″-(3-hydroxy-3-methylglutaroyl)-glucoside]	UV-absorbing agent	[68]
*Glycine max* (L.) Merr.	USA	4′,7-dihydroxyisoflavone, 4′,5,7-trihydroxyisoflavone	Pathogen defense agent	[28]
*Origanum Vulgare* L.	India	Caffeic acid, rosmarinic acid	Pathogen defense agent	[69]
*Apocynum venetum* L.	China	Anthocyanin	Pathogen defense agent	[71]
Edible beans (legumes)	China	Catechin, ferulic acid, protocatechuic acid, gallic acid, p-coumaric acid	Detoxifying agent	[79]
*Heptapleurum arboricola* Hayata	Japan	Quercetin, kaempferol, quercetin glycoside, kaempferol glycoside	Detoxifying agent	[44]
*Ligustrum vulgare* L.	Japan	Quercetin, luteolin	Detoxifying agent	[41]
*Vitis vinifera* L.	Chile	Quercetin, kaempferol	Detoxifying agent	[80]
*Vigna radiata* (L.) R. Wilczek	Japan	Myricetin, quercetin, kaempferol	Detoxifying agent	[42]
*Zea mays* L.	USA	Kaempferol	Fertility of pollen grains, pollination success	[85]
*Petunia hybrida* E. Vilm.	USA	Flavonol aglycones	Fertility of pollen grains	[87]
*Nicotiana tabacum* L.	India	Quercetin	Fertility of pollen grains	[39]
*Solanum lycopersicum* L.	The Netherlands	Flavonoids	Pollination success	[38]

## Data Availability

Not applicable.
